# Establishing a long-term predictive model for aggressive behavior in schizophrenia: a 21-year longitudinal study in rural China

**DOI:** 10.3389/fpsyt.2025.1586009

**Published:** 2025-08-14

**Authors:** Hui Jin, Cong Wang, Yi-Yue Yang, Lie Zhou, Yun Xiao, Yang Wen, Jawad Ahmad, Yun-Fei Mu, Jia Cai, Ming Li, Wei Luo, Xiao-Fei Zhou, Jian-Jun Luo, Bo Liu, Eric Yu-Hai Chen, Mao-Sheng Ran

**Affiliations:** ^1^ Mental Health Center, West China Hospital, Sichuan University, Chengdu, Sichuan, China; ^2^ Department of Social Psychiatry, West China Hospital, Sichuan University, Chengdu, Sichuan, China; ^3^ Xinjin Second People’s Hospital, Chengdu, Sichuan, China; ^4^ Mental Health Center of Chengdu, Chengdu, Sichuan, China; ^5^ Chongqing Mental Health Center, Chongqing, China; ^6^ Mental Health Center of Yangtze University, Jingzhou, Hubei, China; ^7^ National Center of Excellence in Youth Mental Health, University of Melbourne, Melbourne, VIC, Australia

**Keywords:** aggressive behavior, schizophrenia, rural China, longitudinal, predictive model

## Abstract

**Background:**

Although identifying factors contributing to aggressive behavior in individuals with schizophrenia is crucial for developing targeted prevention strategies and intervention, most studies were cross-sectional or short-term, and did not take into account the factor of urbanicity. This study aimed to develop a predictive model of aggressive behavior in individuals with schizophrenia in rural China.

**Method:**

A total of 205 individuals with schizophrenia who were identified in 1994 and followed up in 2015 were included in the study. Aggressive behavior was assessed using the Modified Overt Aggression Scale (MOAS). The final predictive model was developed by backward stepwise regression. The model’s predictive performance was evaluated using the C statistic and calibration curve.

**Result:**

The rate of aggressive behavior in individuals with schizophrenia in rural China was 36.1% during 1994-2015. The final model of aggressive behavior incorporated the following factors: male, lower educational level, unmarried, with delusion, worse social functioning, and with previous treatment. The model demonstrated acceptable discriminative ability, with an AUC of 0.73, sensitivity of 0.82, and specificity of 0.53. The calibration curve indicated a good fit of the model.

**Conclusion:**

The predictive model developed in this study showed good discriminative ability. A clinically practical nomogram was built to assess the risk of aggressive behavior in individuals with schizophrenia in rural China, which may facilitate early detection and intervention of these individuals, particularly in rural areas with limited resources. This approach may be relevant to similar settings internationally.

## Introduction

Schizophrenia is a severe and debilitating mental disorder, with a weighted lifetime prevalence of 0.6% in China (1). It contributes to 13.4 million years of life lived with disability annually, significantly adding to the disease burden (2). Individuals with schizophrenia are at an elevated risk of exhibiting aggressive behavior (3), which leads to a higher care burden for medical staff, extended hospital stays, and increased readmission rates (4). Studies suggest that aggressive behavior in psychiatric settings is often a defensive response to involuntary hospitalization or feeling forced into treatment, rather than intentional violence (5, 6). In addition, command hallucinations may attenuate individuals’ perceived risk of violent behavior (7). Furthermore, limited access to community-based mental health services contribute to the emergence of aggressive behavior (8). Therefore, identifying the risk factors of aggressive behavior in individuals with schizophrenia is crucial for implementing effective interventions and improving long-term outcomes in community-dwelling individuals with schizophrenia.

Aggressive behavior in individuals with schizophrenia is influenced by a combination of clinical, psychological, and social factors (9). Individuals with schizophrenia and their siblings tend to exhibit higher levels of aggressiveness and impulsivity compared to healthy controls, suggesting that familial factors may contribute to the risk of aggressive behavior (10). Hodgins suggested that antisocial traits may play a role in the development of violence (11). However, positive psychotic symptoms, such as persecutory delusions and command hallucinations, are also well-established triggers of aggression (12, 13). Substance use (e.g., alcohol) and experiences of social exclusion or stigma may further increase vulnerability (14, 15). These factors collectively reflect the multifaceted nature of aggressive behavior in schizophrenia.

Most previous studies have utilized a cross-sectional design without follow-up, with individuals with schizophrenia primarily assessed in clinical settings (16–18). Longitudinal studies within a community context, especially rural community remain limited. To address this gap, we utilized data from Chengdu Mental Health Project (CMHP), a 21-year longitudinal follow-up project (1994-2015) in rural China, to explore the most influential factors for long-term aggressive behavior in participants with schizophrenia (19). Our research hypothesis is that the determinants of aggressive behavior in individuals with schizophrenia include not only clinical factors, but also socio-demographical factors.

## Methods

### Participants

Individuals with schizophrenia were identified through the CMHP, which was conducted in March 1994 across six townships in Xinjin County, Chengdu. The survey included a total of 123,572 residents aged 15 years and older. Local households were visited by trained investigators, using the Psychoses Screening Schedule (PSS) and information from local village doctors and community informants (20). Individuals identified as potential patients with psychosis were re-evaluated by psychiatrists using ICD-10 diagnostic criteria. Individuals with schizophrenia were enrolled in this study and subsequently followed up in 2015 (21–25). Informed consent was obtained from all participants. This study was approved by the Human Research Ethics Committee of the University of Hong Kong (HREC no: EA1801025).

### Assessment tools

The Modified Overt Aggression Scale (MOAS) was utilized to assess aggression levels in individuals with schizophrenia, consisting of four subscales: verbal aggression, aggression toward property, self-aggression, and physical aggression toward others. It has showed acceptable reliability and validity in Chinese population (26). Each subscale ranged from 0 (absence of aggression) to 4 (severe aggressive behavior), with higher scores indicating more serious aggressive behavior. Each subscale was weighted as follows: verbal aggression (weight 1), aggression toward property (weight 2), self-aggression (weight 3), and physical aggression (weight 4). The total MOAS score was calculated during the survey in 2015, individuals with schizophrenia with a total score of 5 or higher were categorized into aggressive group (27). The Social Disability Screening Schedule (SDSS) was used to evaluate the social function, which was a 10-item instrument for assessing social disability (28). Each item in this scale was ranged from 0 to 2, where higher scores indicating more serious function obstacle. The SDSS has shown robust reliability and validity in Chinese population (29).

### Assessments

All data were collected through interviews conducted by trained psychiatrists with each subject and his/her family members. Remission status was classified into three categories: complete remission, partial remission and no remission. Delusion was defined as the presence of delusion. Auditory hallucination was defined as the presence of auditory hallucination. Other hallucinations were defined as the presence of visual hallucinations, gustatory hallucinations, and olfactory hallucinations. Marital status was defined as unmarried and married. Education level was categorized as equal or below primary school or above primary school. Psychosocial events were defined as the occurrence of significant life events including death or accident of a close family member, marital conflict, financial loss, setbacks in academic or employment pursuits, and etc. Missing data were handled using the Multiple Imputation method.

### Statistical analysis

The differences of sociodemographic and clinical characteristics and aggressive behaviors from 1994 to 2015 were evaluated, using t tests for continuous variables and chi-square tests for categorical variables. Continuous variables were described as mean and standard deviation, and categorical variables were described as proportions. Predictive factors associated with aggressive behavior were examined using a backward stepwise regression method. The final predictive model was selected based on the Akaike Information Criterion (AIC). Furthermore, a nomogram was created to visualize the model, and calibration was evaluated using Hosmer-Lemeshow test. Results with p value less than 0.05 were considered statistically significant. All analyses were performed by R software (R 4.3.1, packages such as pROC, VRPM, rmda, and ggplot2 were used).

## Results

**Figure 1:**
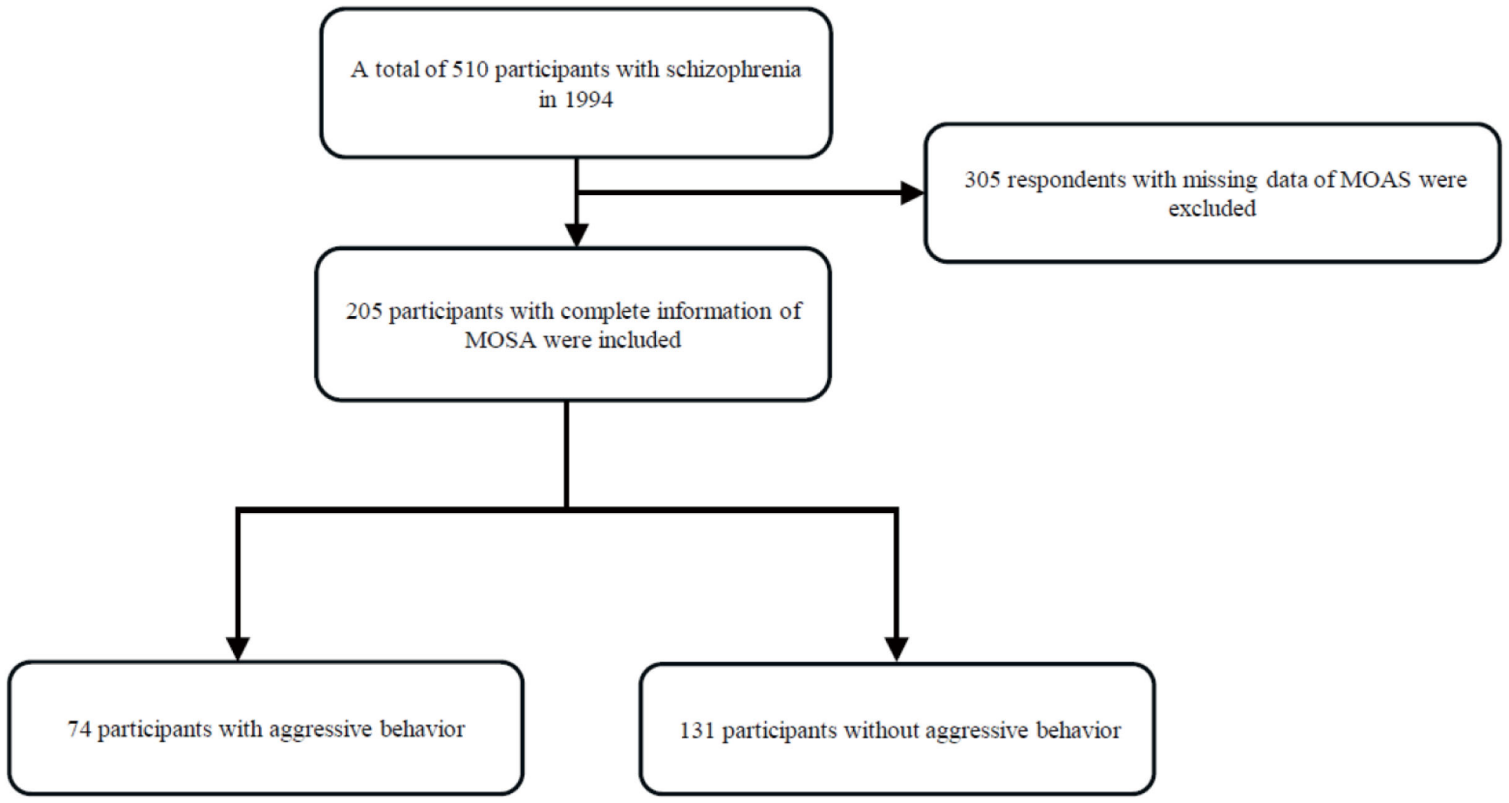
Participant selection for the study.

The selection of participants was shown in **Figure 1**. 510 participants with schizophrenia were identified using the International Classification of Diseases, Tenth Revision (ICD-10) criteria in 1994. 305 participants with schizophrenia died or lost contact. Finally, a total of 205 participants with schizophrenia were followed up in 2015 and included in this study, with 131 (63.9%) participants with non-aggressive behavior and 74 (36.1%) participants with aggressive behavior. The baseline sociodemographic characteristics (**Table 1**) indicated a higher percentage of individuals with aggressive behavior was male (52.7% *vs*. 37.4%, p = 0.05), and unmarried (37.8% *vs*. 22.9%, p = 0.03). No significant differences were observed in terms of age, education level, lifetime suicide attempts, work ability, social functioning, and family history of schizophrenia.

**Table 1:** Baseline sociodemographic characteristics of participants with schizophrenia in 1994.

Characteristics	Non-aggressive (N=131)	Aggressive (N=74)	P.value
Age			
Mean (SD)	41.4 (11.0)	40.6 (13.0)	0.69
Gender			
Man	49 (37.4%)	39 (52.7%)	0.05
Woman	82 (62.6%)	35 (47.3%)	
Education level			
> primary school	41 (31.3%)	14 (18.9%)	0.08
<= primary school	90 (68.7%)	60 (81.1%)	
Marriage			
Married	101 (77.1%)	46 (62.2%)	0.03
Unmarried	30 (22.9%)	28 (37.8%)	
Lifetime suicide attempts			
Yes	12 (9.2%)	6 (8.1%)	1.00
No	119 (90.8%)	68 (91.9%)	
Wok ability			
Have work ability	77 (58.8%)	33 (44.6%)	0.12
Partial work ability	38 (29.0%)	31 (41.9%)	
Without work ability	16 (12.2%)	10 (13.5%)	
Social functioning			
Mean (SD)	5.41 (5.92)	7.27 (6.91)	0.05
Psychosocial events			
No	73 (55.7%)	41 (55.4%)	1.00
Yes	58 (44.3%)	33 (44.6%)	
Family history of schizophrenia			
No	98 (74.8%)	51 (68.9%)	0.46
Yes	33 (25.2%)	23 (31.1%)	
Family population			
Mean (SD)	3.51 (1.41)	3.61 (1.42)	0.64
Income			
High level	14 (10.7%)	6 (8.1%)	0.76
Middle level	52 (39.7%)	28 (37.8%)	
low level	65 (49.6%)	40 (54.1%)	
Caregivers			
without caregivers	8 (6.1%)	3 (4.1%)	0.76
with caregivers	123 (93.9%)	71 (95.9%)	

The clinical characteristics (**Table 2**) showed that delusions were significantly more prevalent in individuals with aggressive behavior compared to those without aggressive behavior (59.5% *vs*. 43.5%, p = 0.04). Other symptoms, including auditory hallucinations, negative symptoms (e.g., poverty of thought and social withdrawal), and treatment status, were not significantly different between the two groups.

**Table 2:** Baseline clinical characteristics of participants with schizophrenia in 1994.

Characteristics	Non-aggressive (N=131)	Aggressive (N=74)	P.value
Clinical characteristics			
Age at first onset (years)			
Mean (SD)	28.7 (9.06)	28.3 (10.7)	0.82
Duration of illness (years)			
Mean (SD)	10.8 (10.5)	11.4 (11.0)	0.71
Treatment status			
Never treated	38 (29.0%)	15 (20.3%)	0.23
Once treated	93 (71.0%)	59 (79.7%)	
Lifetime depressive symptoms			
Yes	62 (47.3%)	44 (59.5%)	0.13
No	69 (52.7%)	30 (40.5%)	
Current mental status			
Complete remission	44 (33.6%)	19 (25.7%)	0.27
Partial remission	19 (14.5%)	8 (10.8%)	
Marked symptoms or deteriorated	68 (51.9%)	47 (63.5%)	
Positive symptoms			
Delusion			
No	74 (56.5%)	30 (40.5%)	0.04
Yes	57 (43.5%)	44 (59.5%)	
Auditory hallucination			
No	80 (61.1%)	39 (52.7%)	0.31
Yes	51 (38.9%)	35 (47.3%)	
Other hallucinations			
No	93 (71.0%)	55 (74.3%)	0.73
Yes	38 (29.0%)	19 (25.7%)	
Hostility			
No	122 (93.1%)	65 (87.8%)	0.30
Yes	9 (6.9%)	9 (12.2%)	
Negative symptoms			
Poverty of thought			
No	95 (72.5%)	54 (73.0%)	1.00
Yes	36 (27.5%)	20 (27.0%)	
Social withdrawal			
No	80 (61.1%)	46 (62.2%)	1.00
Yes	51 (38.9%)	28 (37.8%)	
Blunted affect			
No	94 (71.8%)	51 (68.9%)	0.79
Yes	37 (28.2%)	23 (31.1%)	
Slowed speech			
No	103 (78.6%)	61 (82.4%)	0.64
Yes	28 (21.4%)	13 (17.6%)	

The final predictive model for aggressive behavior included the following factors: gender, education level, social functioning, delusions, marital status, and treatment status (**Table 3**). The model demonstrated that male gender (OR = 1.81, ref: female), lower education level (OR = 2.53, ref: > primary school), social functioning (OR = 1.06), presence of delusions (OR = 2.71, ref: no delusion), unmarried status (OR = 2.10, ref: married), and with previous treatment (OR = 2.76, ref: never treated) were predictors for long-term risk of aggressive behavior.

**Table 3:** Final predictive model of aggressive behavior through stepwise regression.

Groups		OR
Gender	Woman	ref
	Man	1.81
Education level	> primary school	ref
	<= primary school	2.53
Social functioning		1.06
Delusion	No	ref
	Yes	2.71
Marriage	Married	ref
	Unmarried	2.10
Treatment status	Never treated	ref
	Once treated	2.76

OR, Odds ratio.

The predictive factors were categorized into three groups: demographic factors (gender, marital status, educational level), clinical factors (treatment status, presence of delusion), and social factors (social function). We then compared the predictive performance of models that incorporated these factors. Model 1, which included only demographic factors, demonstrated an AUC of 0.62, a sensitivity of 0.47, and a specificity of 0.71. Model 2, which added clinical factors, showed improved predictive performance with an AUC of 0.70, a sensitivity of 0.64, and a specificity of 0.70. Model 3, incorporating social factors in addition to demographic and clinical factors, achieved the highest AUC of 0.73 and sensitivity of 0.82, though its specificity slightly decreased to 0.53 (**Table 4**). These results suggest that incorporating clinical and social factors enhances the model’s predictive ability.

**Table 4:** Comparison of predictive ability between different models.

Parameters	Model 1	Model 2	Model 3
AUC	0.62	0.70	0.73
sensitivity	0.47	0.64	0.82
specificity	0.71	0.70	0.53

Model 1 included demographic factors: gender, marital status, educational level; Model 2 plus clinical factors: treatment status, the presence of delusion; Model 3 plus social factors: social function.

AUC, area under the receiver operator curve.

**Figure 2:**
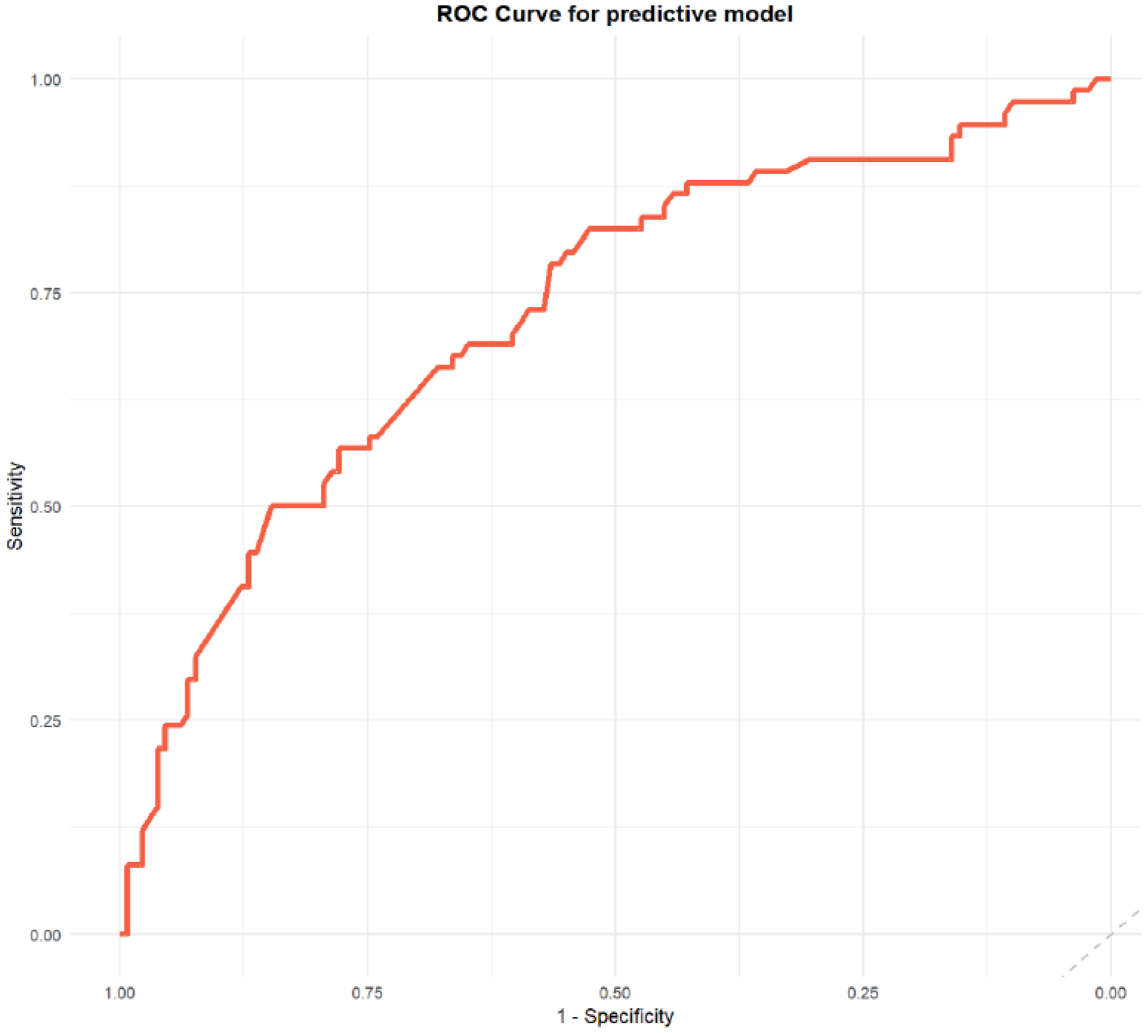
The receiver operating characteristic (ROC) curve for predictive model.

**Figure 3:**
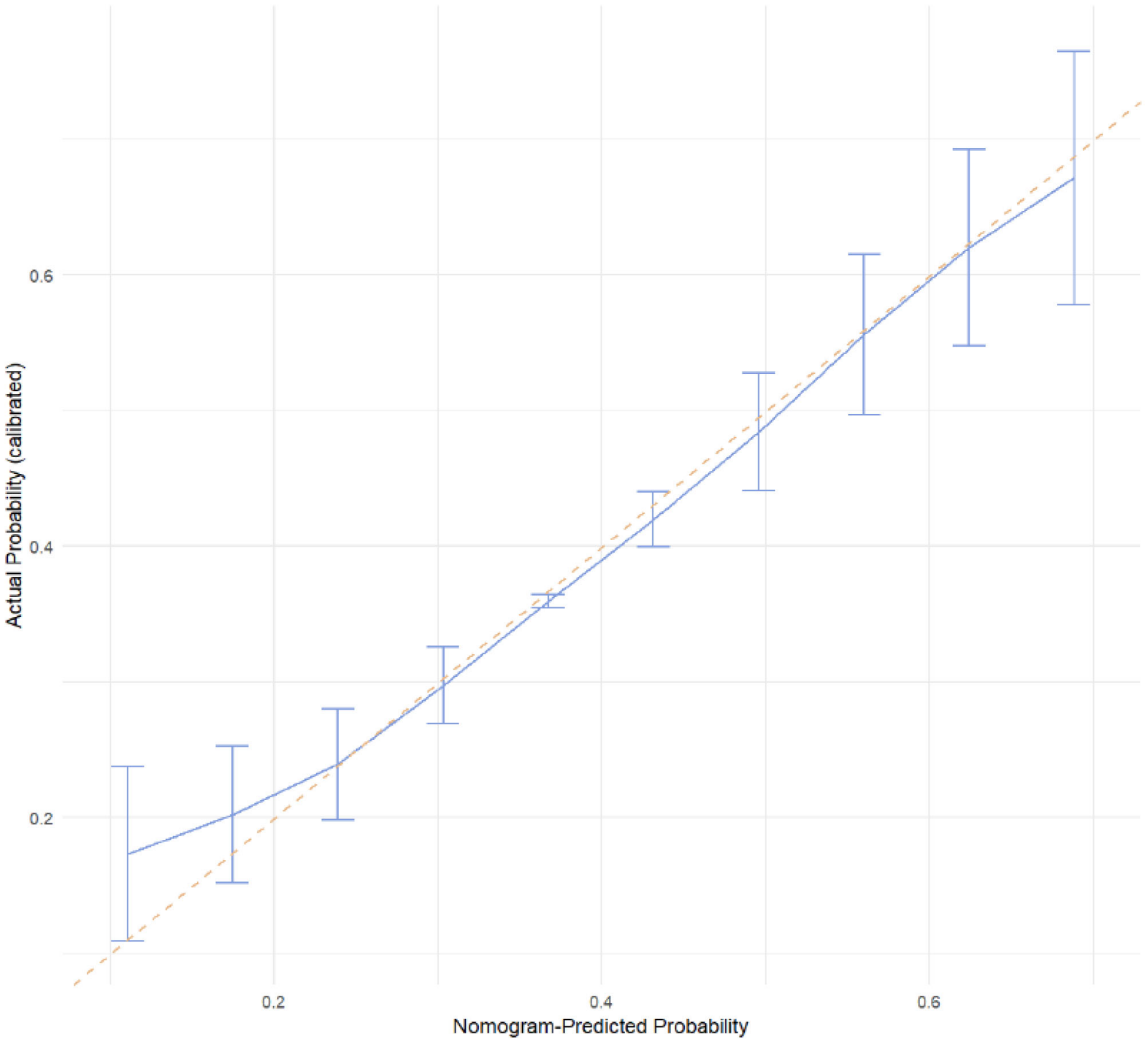
The calibration curve for predictive model.

**Figure 4:**
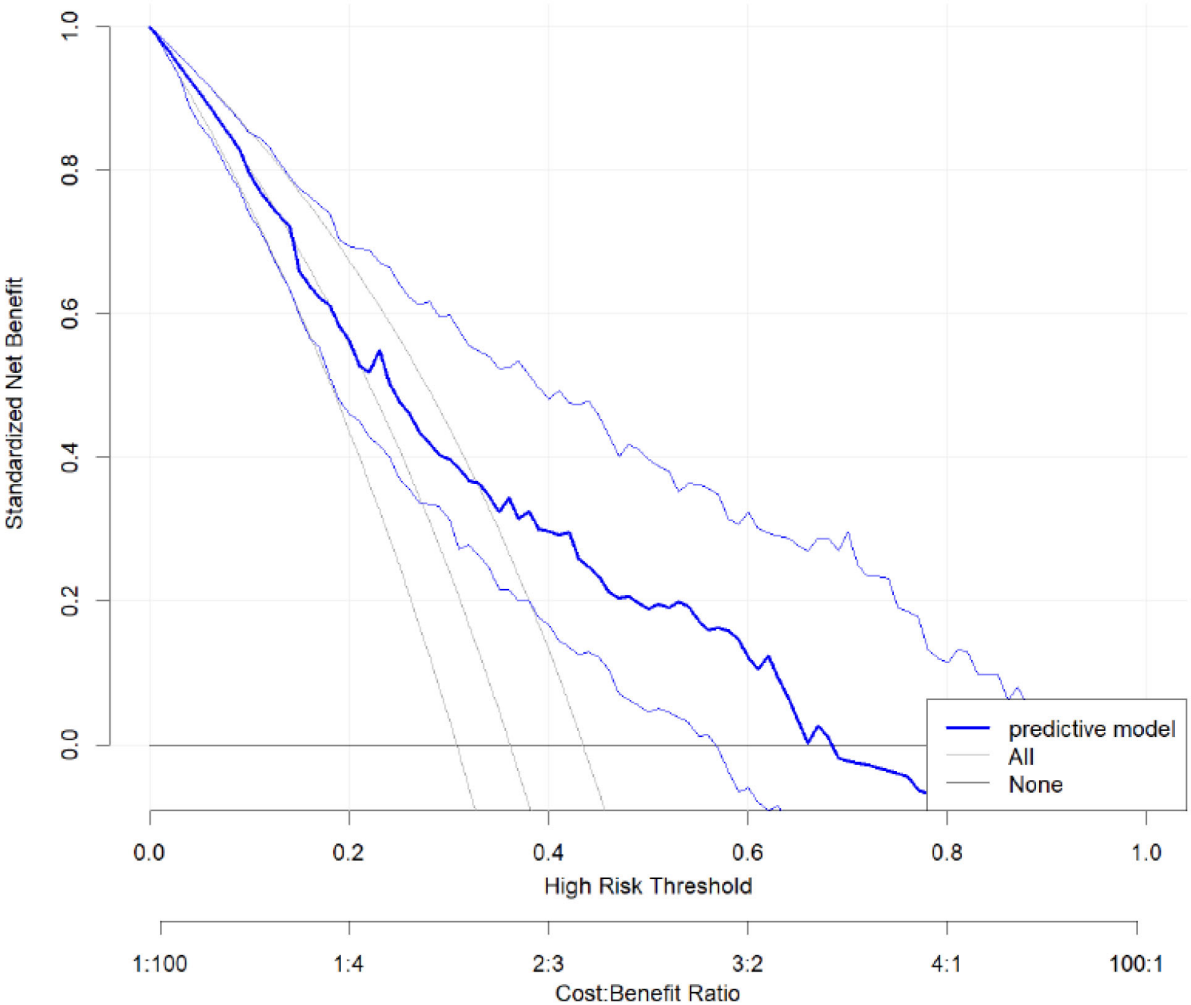
The decision curve analysis for predictive model.

**Figure 5:**
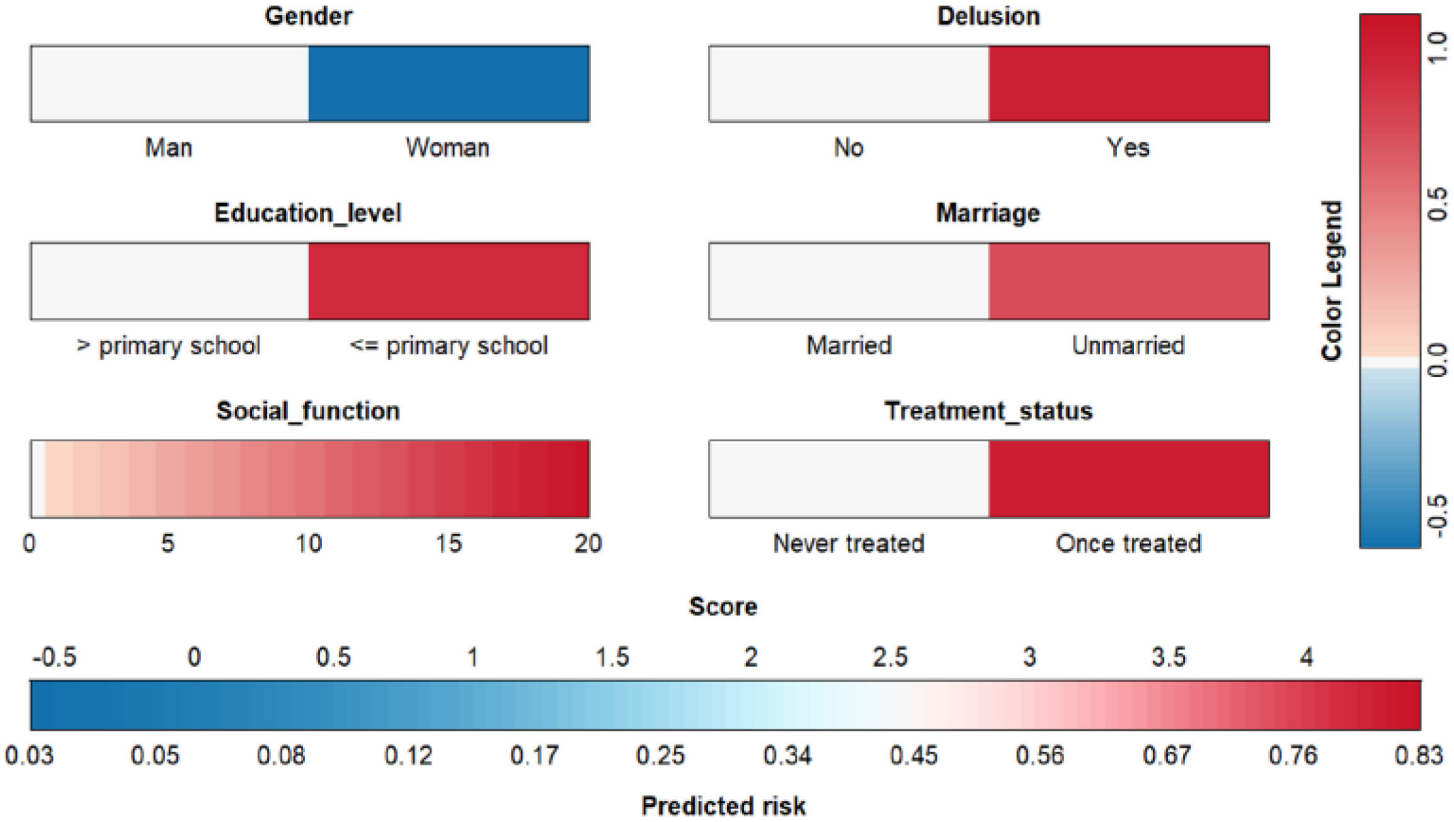
Nomogram for predicting aggressive behavior risk in patients with schizophrenia.

The receiver operating characteristic (ROC) curve (**Figure 2**) showed good discrimination ability for the model, with an area under the curve (AUC) of 0.73. The calibration curve (**Figure 3**) demonstrated a high degree of calibration. The decision curve analysis (DCA) (**Figure 4**) indicated that the model had a higher net benefit compared to the “all” or “none” strategies across the 0.3–0.7 range of risk thresholds. A nomogram was also developed to estimate the probability of aggressive behavior, with each variable assigned a specific point value, and the total score was calculated by summing these points (**Figure 5**). The model exhibited good calibration (Hosmer-Lemeshow test, P = 0.61) and an acceptable discriminative ability with an AUC of 0.73, sensitivity of 0.82, and specificity of 0.53 (**Figure 4**). These findings indicated that the model had clinical utility for identifying individuals at higher risk of aggressive behavior.

## Discussion

The current study has established a predictive model of aggressive behavior among individuals with schizophrenia in rural China. The results of this study showed that the rate of aggressive behavior was 36.1%, which is closely aligned with the results of a meta-analysis of hospitalized individuals in China (35.4%) (30), but significantly higher than the pooled prevalence reported in most western countries, which ranged from 3% to 15% (31). Our study employed a 21-year longitudinal design, which was limited in previous studies on aggressive behavior. Wootton et al. conducted the first longitudinal research with a two-year follow-up (32), while our study firstly revealed long-term predictive factors of aggressive behavior for individuals with schizophrenia over a 21-year time span. The 21-year follow-up period represents a key strength of our study. The included predictors, such as education level, gender, and symptom presentation, are relatively stable over time and can be assessed early in the course of illness. These findings enhance the model’s utility for early risk stratification and support its application in community care for schizophrenia.

Compared to other studies, our research presents a predictive model with good discriminative ability (33). The final model included six key factors: gender, education level, social function, treatment status, marital status, and presence of delusions. Additionally, the model showed good discriminative ability and high consistency between predicted and observed outcomes. Furthermore, we developed a clinically practical nomogram to facilitate the assessment of community-dwelling individuals with schizophrenia.

Evidence shows that social functioning has been considered as important outcome of schizophrenia (34). Worse psychosocial function was related to more serious trouble with the police or with the law in people with mental disorder (35). Yu et al. has proposed significant relationship between SDSS scores and aggressive behavior in a purely male sample with machine learning methods (36). Our study included the results of both males and females. A previous study conducted in China highlighted that other sociodemographic factor, such as childhood trauma and resilience, were significant risk factors of aggressive behavior (37). Correspondingly, a study conducted in Ethiopia showed that poor social support increased the risk of aggressive behavior (adjusted OR = 3.11, 95% CI (1.35, 7.17)) (16). The results of this study also showed that better social functioning was associated with a lower risk of long-term aggressive behavior among individuals with schizophrenia. In line with this, Darmedru et al. reported that cognitive remediation and social cognitive training effectively reduced violence by improving executive and interpersonal skills, highlighting the value of enhancing social functioning in individuals with schizophrenia (38).

Our study had identified male gender as a risk factor, which was in line with our previous study which reported that male gender was associated with criminal behavior (39). In a study conducted in Germany from 1990 to 1995, Soyka et al. reported that the conviction rate was 17.1% in males while only 5.3% in females (40). Furthermore, Wootton et al. found that male gender was a significant predictive factor for the assault over a two-year period in individuals with psychosis (32). Besides, our study highlighted the critical role of higher education level in the prevention of aggressive behavior. Previous study indicates that improving education has been confirmed to reduce the risk of schizophrenia (41). Through education, people can generate socioeconomic resources and solve problems, thus may hinder their aggressive behaviors (42). Increased educational attainment may help mitigate executive deficits in individuals with chronic schizophrenia, thereby reducing their behavioral and functional issues related to the illness (43).

The results of this study showed that marriage was also a significant predictor of aggressive behavior (39). Our previous research demonstrated that unmarried schizophrenics were more likely to show higher level of psychiatric symptoms, lower rate of illness remission, less caregiver, and poorer financial status (44). Research also shows that marriage may deter criminal activity and deviant behavior (45). Swanson et al. found that being single was a predictor of violent behavior within a one-year period in patients with severe mental illness (46). Sampson and Laub proposed that marital attachment increases the likelihood of an offender ceasing criminal involvement (47). Furthermore, interactions between genes and marriage were found to be significant among men (48). Therefore, unmarried status is an important sociodemographic determinant of the occurrence of aggressive behavior. A stable marriage of individuals with schizophrenia may reduce the risk of aggressive behavior in the long term.

The results of this study showed that the presence of delusion was a predictor of aggressive behavior, consistent with previous studies (16, 30). Nielssen et al. highlighted that untreated psychosis was a major contributor to violence in schizophrenia, with inpatient violence significantly associated with psychotic symptoms (49). Zavradashvili et al. also reported that the diagnosis of delusional disorder and the presence of persecution were linked to higher risk of violent acts (28.7% *VS* 7.5%) (50). The possible explanation is that aggressive behavior often arises as a response to psychotic experience, especially delusions (7). Regarding treatment status, the results of this study indicated that a history of treatment was significantly associated with an increased risk of aggressive behavior. This may be because, individuals with schizophrenia who were treated might have more severe symptoms including aggressive behavior (51). Although studies have shown that antipsychotic treatment could reduce both the severity and frequency of aggressive events, some researchers argue that this reduction may occur independently of the antipsychotic effects per se (52). However, further research is needed to better understand this relationship.

Apart from sociodemographic factors, there is also evidence that altered neurobiological processes contribute to aggressive behavior aggressive behavior (53). Studies found that inflammatory markers, such as the monocyte/high-density lipoprotein ratio were significantly elevated in patients with aggressive behavior (54). Additionally, levels of complement component 3 and interleukin-17 were positively correlated with MOAS scores (55). In terms of genetic research, Lachman et al. were the first to report the relationship between catechol-O-methyltransferase (COMT) polymorphism and history of violent behavior (56). Homicidal behavior in schizophrenia has been more frequently observed among individuals carrying the low-activity COMT allele (57). Neuroimaging studies further reveal that patients with schizophrenia and violence exhibited reduced gray matter volume and abnormal brain function in cognitive and emotional tasks (58).

Given the identified risk factors, integrating predictive models with evidence-based strategies represents a promising direction for clinical implementation. For instance, Bussahong et al. demonstrated that a multi-component aggression management program based on behavioral principles significantly reduced the risk of violence in male patients with schizophrenia (59). In addition, Haddock et al. demonstrated that cognitive-behavioral therapy significantly reduced violence in individuals with psychosis and a history of aggression (60). Other psychosocial approaches, such as social skills training, have also shown positive effects in reducing violent behavior by enhancing social participation (61). These findings support combining predictive models with psychosocial interventions to improve the long-term outcome of high-risk patients, especially in rural areas with limited resources.

This study has several strengths. First, it incorporates a wide range of sociodemographic and clinical variables, which significantly enhances the robustness of research findings. Second, to the best of our knowledge, this study represents the first longest follow-up research on aggressive behavior among individuals with schizophrenia in rural China. It serves as a valuable reference for other developing countries facing similar conditions. Furthermore, given the limited mental health facilities and professionals in rural China, we have developed an efficient and practical predictive model for identifying individuals with schizophrenia at risk of aggressive behavior in community settings. This tool enables a low-cost identification of aggressive behavior. For those identified as high risk, more immediate and effective interventions can be implemented to prevent aggressive incidents.

This study also has some limitations. First, due to the relatively small sample size, the conclusions drawn may not be fully reliable. Second, the absence of information on substance use in the 1994 survey prevents us from accounting for their potential influence on our results. In the 2015 follow-up, substance use was formally assessed. Two participants in each group (aggressive and non-aggressive) reported a history of alcohol use, and no other forms of substance use were identified. Only one participant met the diagnostic criteria for substance dependence and did not exhibit aggressive behavior. This low prevalence may reflect the strict regulation of illicit drugs in China, which contrasts with patterns observed in many Western countries (62). Third, the predictive model lacks external validation, which limits the generalizability of our findings. Finally, given the sociocultural and developmental differences, the results of this study may not be applicable to high-income countries.

## Conclusion

The rate of aggressive behavior among individuals with schizophrenia in rural China is 36.1% during a 21-year follow-up period. Our study has developed a predictive model for aggressive behavior in individuals with schizophrenia, incorporating the following factors: male, lower educational level, unmarried, with delusion, worse social functioning, and with previous treatment. The model showed good discriminative ability. A nomogram was built to easily estimate aggressive behavior, which may facilitate in early intervention.

## Data Availability

The data analyzed in this study is subject to the following licenses/restrictions: The datasets generated and analyzed during the current study are available from the corresponding author on reasonable request. Requests to access these datasets should be directed to msrancd@outlook.com.
